# Effect of fracturoscopy on the incidence of surgical site infections post tibial plateau fracture surgery

**DOI:** 10.1007/s00068-020-01486-y

**Published:** 2020-09-15

**Authors:** Ralf Henkelmann, Matthias Krause, Lena Alm, Richard Glaab, Meinhard Mende, Christopher Ull, Philipp-Johannes Braun, Christoph Katthagen, Tobias J. Gensior, Karl-Heinz Frosch, Pierre Hepp

**Affiliations:** 1 Department of Orthopedics, Trauma and Plastic Surgery, University of Leipzig, Liebigstraße 20, 0410 Leipzig, Germany; 2 Clinic of Trauma, Hand and Reconstructive Surgery, University Medical Center Hamburg-Eppendorf, Hamburg, Germany; 3 Division of Knee and Shoulder Surgery, Department of Trauma and Reconstructive Surgery, Sports Traumatology, Asklepios Klinik St. Georg, Hamburg, Germany; 4 Centre for Clinical Trials, University of Leipzig, Leipzig, Germany; 5 Clinic for Arthroscopic Surgery, Sports Traumatology and Sports Medicine, BG Clinic, Duisburg, Germany; 6 Department of General and Trauma Surgery, BG University Hospital Bergmannsheil, Bochum, Germany; 7 Department of Trauma and Orthopaedic Surgery, BG Hospital Unfallkrankenhaus Berlin gGmbH, Berlin, Germany; 8 Department of Trauma, Hand and Reconstructive Surgery, University Hospital Münster, Münster, Germany; 9 Department of Traumatology, Cantonal Hospital Aarau, Aarau, Switzerland; 10 Orthopädische Praxisklinik Neuss-Düsseldorf (OPND), Düsseldorf, Germany

**Keywords:** Tibial plateau fracture, Surgical site infection, Fracturoscopy, ARIF, ORIF, Postoperative infection

## Abstract

**Purpose:**

Surgical treatment of tibial plateau fracture (TPF) is common. Surgical site infections (SSI) are among the most serious complications of TPF. This multicentre study aimed to evaluate the effect of fracturoscopy on the incidence of surgical site infections in patients with TPF.

**Methods:**

We performed a retrospective multicentre study. All patients with an AO/OTA 41 B and C TPF from January 2005 to December 2014 were included. Patients were divided into three groups: those who underwent arthroscopic reduction and internal fixation (ARIF), and those who underwent open reduction and internal fixation (ORIF) with fracturoscopy, and those treated with ORIF without fracturoscopy. The groups were compared to assess the effect of fracturoscopy. We characterised our cohort and the subgroups using descriptive statistics. Furthermore, we fitted a logistic regression model which was reduced and simplified by a selection procedure (both directions) using the Akaike information criterion (AIC). From the final model, odds ratios and inclusive 95% confidence intervals were calculated.

**Results:**

Overall, 52 patients who underwent fracturoscopy, 48 patients who underwent ARIF, and 2000 patients treated with ORIF were identified. The rate of SSI was 0% (0/48) in the ARIF group and 1.9% (1/52) in the fracturoscopy group compared to 4.7% (93/2000) in the ORIF group (OR = 0.40, *p* = 0.37). Regression analyses indicated a potential positive effect of fracturoscopy (OR, 0.65; 95% CI, 0.07–5.68; *p* = 0.69).

**Conclusion:**

Our study shows that fracturoscopy is associated with reduced rates of SSI. Further studies with larger cohorts are needed to investigate this.

**Level of evidence:**

Level III.

## Introduction

The surgical treatment of tibial plateau fractures (TPF) is common and surgical site infections (SSI) remain one of the most serious complications [1]. The rate of SSI can be reduced by interval surgical treatment, fracture specific surgical approaches, and less invasive techniques [2]. Additionally, the surgery duration and intraoperative blood loss have been identified as independent risk factors for SSI [3].

Recently, fracturoscopy has been used in TPF to help improve intraoperative fracture reduction [4–7]. Furthermore, different approaches for fracturoscopy have been reported. Krause et al. inserted the arthroscope directly via the surgical approach to achieve visualisation of the fracture which is also known as fracturoscopy [4]. Fracturoscopy is characterised by low or no intra-articular water pressure due to direct arthroscopic imaging of the fracture. Therefore, it is not an arthroscopic reduction and internal fixation (ARIF) in the true sense since the capsule is opened via a larger approach [5, 7–9]. This multicentre study aimed to evaluate the influence of direct fracturoscopy on the incidence of SSIs in patients surgically treated for tibial plateau fractures.

## Methods

We conducted a retrospective multicentre study at 7 level-1 trauma centres in Germany and Switzerland. This study was performed in line with the principles of the Declaration of Helsinki. The study was approved by the leading ethical committee (Reference number: 098/15-ff) and by the corresponding ethics committees of all the participating hospitals. The requirement for informed consent from patients was waived due to the retrospective nature of the study. Both ARIF (arthroscopic reduction and internal fixation) and ORIF (open reduction and internal fixation with or without fracturoscopy) (Hamburg, Leipzig, Aarau) were performed at three centres while ORIF with or without fracturoscopy was performed at four centres (Duisburg, Bochum, Münster, Berlin). In contrast to ARIF, with arthroscopy applied in conventional ways and a typical water pressure between 35 and 55 mmHg, fracturoscopy does not require increased water pressure as it is introduced into the joint through an open surgical approach.

### Participants

All patients who were surgically treated for proximal tibia fractures in the participating hospitals from January 2005 to December 2014 were identified by querying the hospitals’ databases using the International Classification of Disease (ICD) code for tibial plateau fractures. The medical record of each patient was manually screened to avoid the inclusion of patients who were improperly coded, were primarily operated on in another hospital or did not fit in our inclusion criteria.

The inclusion criteria were as follows: age > 18 years, primarily treated in a participating hospital, and proximal tibial fracture classified as AO 41 B or C. Exclusion criteria were as follows: AO 41 A fractures and primary treatment with arthroplasty. The cohort was divided into three groups: ARIF, fracturoscopy, and ORIF.

### Variables

In addition to standard parameters (age, sex, body mass index), pre-existing conditions were categorised into four groups according to the number of comorbidities: no comorbidity, 1–3 comorbidities, 4–5 comorbidities, and ≥ 6 comorbidities. Diabetes mellitus, nicotine abuse, alcohol/drug abuse, as well as intake of immunosuppressive drugs were listed separately at the nominal scale level. Other concomitant injuries were categorised as none, not relevant (haematoma, abrasions, grade 1 soft tissue damage according to Gustilo and Anderson, grade 1 craniocerebral trauma), and relevant (fracture to other body regions, grade > 1 soft tissue damage according to Gustilo and Anderson, grade > 1 craniocerebral trauma) [10]. Patients with an Injury Severity Score (ISS) > 16 were classified as having those with polytrauma [11].

Fracture morphology was classified according to the AO/OTA Fracture and Dislocation Classification [12]. We recorded the following variables: open fracture, compartment syndrome, administration of blood bags, and time of operation (day = 08:00 a.m. to 08:00 p.m., night = 08:00 p.m. to 08:00 a.m.).

SSI were recorded according to the definition proposed by the current protocol of the National Healthcare Safety Network, Centres for Disease Control and Prevention [13–15]. This definition is used in the German guideline of the Robert Koch Institute as well as by the World Health Organisation.

### Statistical analysis

We characterised our cohort and the subgroups using descriptive statistics: Mean (standard deviation, SD) for continuous variables and number (%) for categorical variables. As a measure for the association between the groups with/without fracturoscopy, the standardised mean difference (SMD) was calculated for continuous variables and odds ratio for binary variables.

Further, we examined if fracturoscopy remained a relevant and significant factor when the effect is adjusted by known covariates. With this aim, we fitted a binary logistic regression model for infection with fracturoscopy and 13 additional baseline and peri-operative covariates. The model was reduced and simplified by a selection procedure (both directions) using the Akaike information criterion (AIC). From the final model, odds ratios including 95% confidence intervals were calculated.

Data preparation and descriptive statistics were analysed using IBM SPSS Statistics, version 26. Using the R software, multiple models were fitted, and graphs were generated. The significance level was set at 5% for two-tailed testing.

## Results

Of the 2100 patients who underwent surgery for TPF, 100 patients who underwent fracturoscopy (*n* = 52) or ARIF (*n* = 48) were identified. The rate of SSI was 1.9% (1/52) in the fracturoscopy group compared to 0% in the ARIF (0/48) and 4.7% (93/2000) in the ORIF group (fracturoscopy vs. ORIF: OR = 0.40, *p* = 0.37). Tables 1, 2, 3 compare the three groups (ARIF, Fracturoscopy, and ORIF) in terms of descriptive (Table 1), fracture-related (Table 2), and surgery-related (Table 3) variables. The groups were similar in terms of age and weight (Table 1). There were more men in the fracturoscopy group compared to the ARIF and ORIF groups. The groups were similar in terms of comorbidities and the number of patients with diabetes mellitus. There were more smokers in the ARIF and fracturoscopy group while there were more patients with immunosuppression in the ORIF group. There were patients with only B fractures in the ARIF Group (Table 2). A comparison between ORIF and fracturoscopy groups showed more C fractures in the ORIF group (44.8 vs. 28.8%), while the number of patients with concomitant injuries (29.5% vs. 34.5%), polytraumas (11.4% vs. 7.7%), and use of blood bags (9.9% vs. 7.7%) were comparable. Compartment syndrome occurred more frequently in the ORIF group compared to the fracturoscopy group (6.9% vs. 1.9%). Primary surgery in ORIF was performed at night in 44.2% of the cases (fracturoscopy, 1.9%).

**Table 1:** Descriptive patient dependent data

Parameter	Categories	ORIF (*n* = 2000)		ARIF (*n* = 48)		Fracturoscopy (*n* = 52)		SMD
		Mean (SD)	Range	Mean (SD)	Range	Mean (SD)	Range	ORIF vs. fracturoscopy
Age (years)		50.3 (15.1)	18–95	42.8 (13.3)	20–77	48.4 (15.4)	18–82	0.13
Weight (kg)		80 (19)	34–172	78.8 (14.7)	54–120	77.9 (17.7)	47–120	0.11
BMI (kg/m^2^)		26.7 (5.4)	11.4–52	25.9 (4.4)	18–36	25.8 (5)	17.3–39	0.17

Parameter	Categories	ORIF (*n* = 2000)		ARIF (*n* = 48)		Fracturoscopy (*n* = 52)		SMD
		*n*	%	*n*	%	*n*	%	OR
Sex	Male	1087	54.4	24	50.0	32	61.5	1.34
	Female	912	45.6	24	50.0	20	38.5	
Age category	≤ 39 years	475	23.8	21	43.8	16	30.8	1.43
	40–64 years	1184	59.2	25	52.1	29	55.8	
	65–79 years	273	13.7	2	4.2	6	11.5	
	≥ 80 years	68	3.4	0	0.0	1	1.9	
Comorbidities	None	1180	59.5	33	68.8	36	69.2	1.53
	1–3	703	35.4	14	29.2	13	25.0	
	4–5	75	3.8	1	2.1	2	3.8	
	≥ 6	26	1.3	0	0.0	1	1.9	
DM	No/unknown	1877	94.0	48	100.0	47	90.4	1.66
	Yes	120	6.0	0	0.0	5	9.6	
Smoking	No/unknown	1642	82.4	28	59.6	36	69.2	2.08
	Yes	351	17.6	19	40.4	16	30.8	
IS	No/unknown	1964	98.3	48	100.0	52	100.0	–
	Yes	33	1.7	0	0.0	0	0.0	

*BMI* body mass index, *DM* diabetes mellitus, *ORIF* open reduction and internal fixation, *ARIF* arthroscopy reduction and internal fixation, *SD* standard deviation, *SMD* standardised mean difference, *OR* odds ratio, *IS* immunosuppression

**Table 2:** Descriptive trauma dependent data

Parameter	Category	ORIF (*n* = 2000)		ARIF (*n* = 48)		Fracturoscopy (*n* = 52)		OR
		*n*	%	*n*	%	*n*	%	
AO/OTA	B1	143	7.7	10	20.8	3	5.8	
	B2	320	17.3	28	58.3	16	30.8	
	B3	561	30.3	10	20.8	18	34.6	
	C1	183	9.9	0	0.0	3	5.8	
	C2	152	8.2	0	0.0	1	1.9	
	C3	495	26.7	0	0.0	11	21.2	
AO/OTA	B	1024	55.2	48	100.0	37	71.2	
	C	830	44	0	0.0	15	28.8	0.50
Open fracture	No/unknown	1911	95.7	48	100.0	50	96.2	
	Yes	86	4.3	0	0.0	2	3.8	0.89
Compartment syndrome	No/unknown	1862	93.1	48	100.0	51	98.1	
	Yes	138	6.9	0	0.0	1	1.9	0.26
Polytrauma	Yes	1773	88.7	45	93.8	48	92.3	
	No	227	11.4	3	6.3	4,00	7.7	0.65
Relevant concomitant injury	No/unknown	1407	70.5	31	64.6	34	65.4	
	Yes	590	29.5	17	35.4	18	34.6	1.26
Operation Time	Night	884	44.2	0	0.0	1	1.9	
	Day	1116	55.8	48	100.0	51	98.1	40.4
Blood bag use	No/unknown	1802	90.1	46	95.8	48	92.3	
	Yes	198	9.9	2	4.2	4	7.7	0.76

*ORIF* open reduction internal fixation, *ARIF* arthroscopic reduction and internal fixation, *OR* odds ratio

**Table 3:** Descriptive operation dependent data

	ORIF (*n* = 2000)		ARIF (*n* = 48)		Fracturoscopy (*n* = 52)	
	*n*	%	*n*	%	*n*	%
Screw	244	12.2	48	100.0	1	1.9
Plate	861	43.1	0	0.0	27	51.9
External fixation	474	23.7	0	0.0	4	7.7
Double plate	95	4.8	0	0.0	3	5.8
Plate and screw	305	15.3	0	0.0	16	30.8
Other	21	1.1	0	0.0	1	1.9

*ORIF* open reduction internal fixation, *ARIF* arthroscopic reduction and internal fixation

Screw osteosynthesis was used in all the patients treated by ARIF. A comparison between ORIF and fracturoscopy revealed that there were differences in the primary application of the external fixator and the combination of screws and plates. Patients with fracturoscopy and ORIF were most frequently treated by plate osteosynthesis (Table 3). Four patients with a primary external fixator in the fracturoscopy group were treated with a plate (*n* = 1) or a double plate (*n* = 3).

**Fig. 1:**
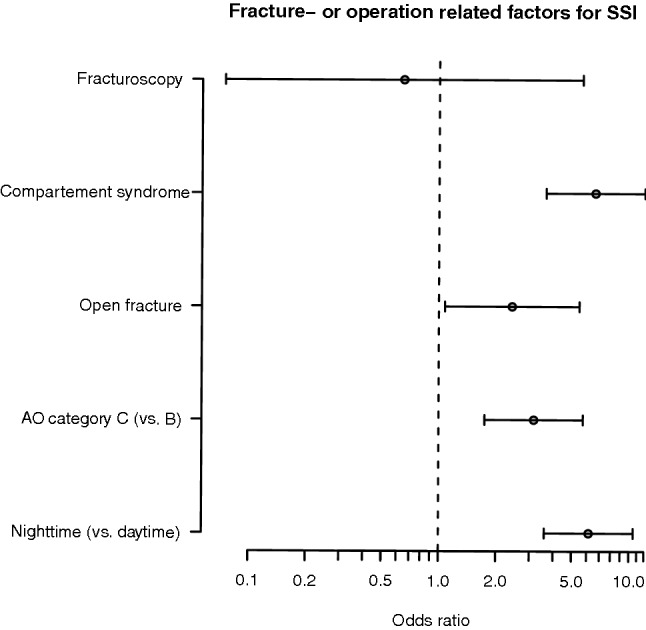
Error bar plot of fracture or operation related factors for SSI

Multiple regression analysis showed an increased risk of SSI related to various factors (Table 4). Furthermore, the positive effect of fracturoscopy associated with the reduction of SSI remained consistent even after adjustment for possible covariates (OR = 0.65; 95% CI, 0.07–5.68; *p* = 0.69). However, only low statistical evidence was observed for this effect likely due to confounding by several covariates that were not possible to control. Figure 1 shows the effect of fracturoscopy depicted in a Forrest plot compared to other fracture- or operation related factors. The other covariates are listed in detail in Table 4.

**Table 4:** Multivariable logistic regression model of potential risk factors of SSI compared between ORIF and fracturoscopy

	OR	95% CI		*p* value
Fracturoscopy	0.65	0.07	5.68	0.69
BMI (per 5 kg/m^2^)	1.35	1.08	1.67	0.006
Smoker (vs. none)	2.16	1.25	3.73	0.005
Immune suppression	5.53	1.59	19.3	0.006
Compartment syndrome	6.64	3.65	121	0.000
Polytrauma	2.05	1.02	4.12	0.039
Open fracture	2.43	1.08	5.47	0.029
Comorbidities				
1–3	1.64	0.93	2.90	0.081
4–5−	6.02	2.47	14.7	0.000
6 +	7.63	2.01	28.9	0.002
AO category (C vs. B)	3.16	1.74	5.73	0.000
Night-time	6.15	3.60	10.5	0.000

The reference category for co-morbidities was ‘none’

*OP* operation, *OR* odds ratio, *CI* confidence interval, *BMI* body mass index

## Discussion

This study evaluated potential factors associated with fracturoscopy that may be good indicators of SSI reduction. Analysis of our data showed that the rate of SSI in the group with fracturoscopy was 1.9% which was substantially lower compared to the ORIF group (4.7%). Furthermore, the regression analysis indicated the positive effect of fracturoscopy on SSI (OR 0.65; 95% CI 0.07–5.68; *p* = 0.69). However, the large confidence interval and the *p *value indicates that this effect is statistically uncertain. This can be traced back to the single case of infection in the fracturoscopy group. To the best of our knowledge, this is the first study to focus on the association between fracturoscopy and SSI. In comparison with previously published studies, it was observed that patients treated by ARIF rarely had an SSI. Verona et al. compared 19 ARIF cases with 21 ORIF cases for Schatzker I–III fractures where one case of SSI was found in the ORIF group [8]. In a retrospective multicentre study, Le Baron et al. [5] compared 77 patients with ARIF and 240 patients with ORIF (Schatzker I–III) where two cases of SSI were found in the ARIF group and four in the ORIF group. Similar results were also reported by Wang et al. with 26 ARIF vs 41 ORIF cases and Elabjer et al. with 40 ARIF vs 38 ORIF cases where one case of SSI was observed in both of the ORIF groups [6, 7]. Our study revealed identical surgical trends and their association with SSI in patients treated by ARIF. However, the surgical technique of fracturoscopy differs significantly from ARIF. This is reflected in the fracture morphology and the osteosynthesis methods used in the fracturoscopy group. Fracturoscopy provides an identical procedure to ORIF for the treatment of complex TPF. Therefore, it is possible to compare fracturoscopy with the studies currently available on ORIF in terms of risk factors for SSI. As previously described in other studies, our study also shows a correlation between patient-dependent factors (e.g. weight, smoking, previous illnesses) and fracture- or operation-dependent factors (e.g. fracture morphology, compartment syndrome, polytrauma) [16–18]. Furthermore, the exact mechanism on how fracturoscopy has a positive effect on reducing SSI remains unclear; possible factors may include low operative complication rates such as lower blood loss and smaller operative approach with less soft tissue damage. Further studies to clarify these are needed.

## Conclusion

Study results indicate, that fracturoscopy and ARIF might be associated with a reduced rate of SSI in patients surgically treated with TPF. Currently, the number of influential factors associated with SSI reduction in the operative treatment of fractures is limited. Fracturoscopy could play a role and should be re-evaluated with significantly higher case numbers. Fracturoscopy offers obvious advantages intraoperatively, and it now also appears to have a potential advantage postoperatively.

## Limitations

Limitations of the study include its retrospective design. Furthermore, there was an imbalance between the investigated groups regarding group size and also the distribution of variables within the groups. This was addressed with the help of biometric analyses; however, the effect of some potential bias cannot be ruled out. Furthermore, some risk factors (e.g. surgery time, number of incision sites and irrigation volumes) that could influence the occurrence of SSI were not evaluated in this study.

## Data Availability

Data on the study are available on request from the corresponding author.
