# Correction to: Effect of fracturoscopy on the incidence of surgical site infections post tibial plateau fracture surgery

**DOI:** 10.1007/s00068-020-01506-x

**Published:** 2020-10-20

**Authors:** Ralf Henkelmann, Matthias Krause, Lena Alm, Richard Glaab, Meinhard Mende, Christopher Ull, Philipp-Johannes Braun, Christoph Katthagen, Tobias J. Gensior, Karl-Heinz Frosch, Pierre Hepp

**Affiliations:** 1 Department of Orthopedics, Trauma and Plastic Surgery, University of Leipzig, Liebigstraße 20, 0410 Leipzig, Germany; 2 Clinic of Trauma, Hand and Reconstructive Surgery, University Medical Center Hamburg-Eppendorf, Hamburg, Germany; 3 Division of Knee and Shoulder Surgery, Department of Trauma and Reconstructive Surgery, Sports Traumatology, Asklepios Klinik St. Georg, Hamburg, Germany; 4 Centre for Clinical Trials, University of Leipzig, Leipzig, Germany; 5 Clinic for Arthroscopic Surgery, Sports Traumatology and Sports Medicine, BG Clinic, Duisburg, Germany; 6 Department of General and Trauma Surgery, BG University Hospital Bergmannsheil, Bochum, Germany; 7 Department of Trauma and Orthopaedic Surgery, BG Hospital Unfallkrankenhaus Berlin gGmbH, Berlin, Germany; 8 Department of Trauma, Hand and Reconstructive Surgery, University Hospital Münster, Münster, Germany; 9 Department of Traumatology, Cantonal Hospital Aarau, Aarau, Switzerland; 10 Orthopädische Praxisklinik Neuss-Düsseldorf (OPND), Düsseldorf, Germany

## Correction to: European Journal of Trauma and Emergency Surgery 10.1007/s00068-020-01486-y

The original version of this article unfortunately contained a mistake. In the author list, the first and last names were tagged incorrectly. The corrected author list is given above.

The original article has been corrected.

